# Hyperpolarization-Enhanced NMR Spectroscopy of Unaltered Biofluids Using Photo-CIDNP

**DOI:** 10.1021/acs.analchem.3c03215

**Published:** 2023-12-18

**Authors:** Lars T. Kuhn, Stefan Weber, Joachim Bargon, Teodor Parella, Míriam Pérez-Trujillo

**Affiliations:** † Institut für Physikalische Chemie, Albert-Ludwigs-Universität Freiburg, Albertstr. 21, 79104 Freiburg i. Br., Germany; ‡ Institut für Physikalische und Theoretische Chemie, Rheinische Friedrich-Wilhelms-Universität Bonn, Wegelerstr. 12, 53115 Bonn, Germany; § Servei de Ressonància Magnètica Nuclear, Facultat de Ciències i Biosciències, Universitat Autònoma de Barcelona, 08193 Cerdanyola del Vallès, Catalonia, Spain

## Abstract

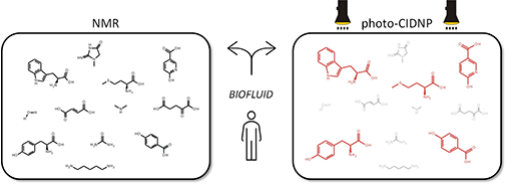

The direct and unambiguous detection and identification of individual metabolite molecules present in complex biological mixtures constitute a major challenge in (bio)analytical research. In this context, nuclear magnetic resonance (NMR) spectroscopy has proven to be particularly powerful owing to its ability to provide both qualitative and quantitative atomic-level information on multiple analytes simultaneously in a noninvasive manner. Nevertheless, NMR suffers from a low inherent sensitivity and, moreover, lacks selectivity regarding the number of individual analytes to be studied in a mixture of a myriad of structurally and chemically very different molecules, e.g., metabolites in a biofluid. Here, we describe a method that circumvents these shortcomings via performing selective, photochemically induced dynamic nuclear polarization (photo-CIDNP) enhanced NMR spectroscopy on unmodified complex biological mixtures, i.e., human urine and serum, which yields a single, background-free one-dimensional NMR spectrum. In doing this, we demonstrate that photo-CIDNP experiments on unmodified complex mixtures of biological origin are feasible, can be performed straightforwardly in the native aqueous medium at physiological metabolite concentrations, and act as a spectral filter, facilitating the analysis of NMR spectra of complex biofluids. Due to its noninvasive nature, the method is fully compatible with state-of-the-art metabolomic protocols providing direct spectroscopic information on a small, carefully selected subset of clinically relevant metabolites. We anticipate that this approach, which, in addition, can be combined with existing high-throughput/high-sensitivity NMR methodology, holds great promise for further in-depth studies and development for use in metabolomics and many other areas of analytical research.

## Introduction

The simultaneous detection and identification of a multitude of chemically and structurally very different analytes present in complex biological mixtures have gained significant relevance in recent years. In particular, the advent of the rapidly growing field of metabolomics research^1,2^ has highlighted the need for automated high-throughput analytical methods that allow the concurrent analysis of multiple metabolites in complex biofluids. In this context, nuclear magnetic resonance (NMR) spectroscopy has emerged as a key analytical technique given its ability to extract extremely precise qualitative and, in many cases, also quantitative information on numerous metabolites simultaneously in a fully noninvasive manner. Thus, the method has found wide applicability in areas such as metabolism studies and metabolic profiling as well as many other disciplines where complex biological mixtures containing, for example, clinically relevant metabolites require efficient characterization.^3^

Despite the advantages NMR offers compared to other analytical platforms, e.g., mass spectrometry, its inherent sensitivity is low. Hence, a variety of different methodological improvements have been developed over the last decades to overcome this drawback. These range from the introduction of higher magnetic fields and cryogenically cooled NMR probes to other, more exotic, setup modifications such as the use of microfluidics and NMR microcoil detection methods as well as combinations thereof.^4^ Another strategy that has been pursued to substantially decrease the detection limit of NMR is the application of so-called hyperpolarization methods, i.e., physical or chemical means to increase the signal-to-noise ratio of an NMR measurement performed in liquids. Among those, dissolution dynamic nuclear polarization (d-DNP)^5^ as well as hydrogenative and non-hydrogenative parahydrogen-induced polarization procedures (PHIP and NH-PHIP)^6^ have featured most prominently in recent years.

Both d-DNP and PHIP have also been employed to facilitate the study of complex biological mixtures. For example, Tessari and co-workers utilized non-hydrogenative PHIP in order to detect and quantify α-amino acids in human urine following dilution of the sample with methanol in the presence of an iridium-based precatalyst.^7^ In another study, Dey et al. presented an untargeted NMR-based metabolomic workflow based on dissolution DNP thereby enabling hyperpolarized ^13^C metabolomics of plant extracts at natural abundance.^8^ Despite these and several other extremely encouraging results,^9,10^ typical liquid-state NMR experiments employing d-DNP- or parahydrogen-based hyperpolarization feature very specific and demanding sample preparation/setup requirements, e.g., long polarization times in combination with the occasional need for expensive additional instrumentation, which render biocompatible and automated high-throughput NMR measurements challenging.

Photochemically induced dynamic nuclear polarization (photo-CIDNP), another nuclear spin-selective technique, uses somewhat milder experimental conditions and involves the instantaneous (low-power) LED- or laser light-induced generation of transient radical pairs in the presence of a small amount of a photosensitizer yielding spin-polarized molecular species.^11−13^ In addition, photo-CIDNP is a very sensitive homo- and heteronuclear hyperpolarization method that allows the NMR detection of analytes present in a low nanomolar concentration range provided that certain experimental requirements are met.^14,15^ Traditionally, the method has been mainly employed to gauge biomacromolecular solvent exposure in the steady state as well as in real-time owing to its ability to selectively highlight the three aromatic amino acid side chains of tyrosine (Tyr), tryptophan (Trp), and histidine (His) as well as the aliphatic amino acid methionine (Met). Thus, up to the present time, biological photo-CIDNP experiments have been conducted almost exclusively for the analysis of isolated amino acids and proteins in buffered aqueous solution. To the best of our knowledge, only one precedent of the application of photo-CIDNP NMR spectroscopy in the context of (complex) biological media has been reported using specific means, which seem difficult to implement into metabolomic workflows: the detection of a singly ^13^C-labeled Trp isotopologue in a diluted bacterial cell extract applying a low concentration (LC) photo-CIDNP approach involving multistep pretreatment procedures of the sample solution prior to acquisition of the hyperpolarization spectrum.^14,15^

Here, we demonstrate the feasibility of conducting liquid-state photo-CIDNP NMR experiments for the direct and very selective analysis of complex biological mixtures, i.e., human urine and serum, in a rapid, minimally invasive manner, thereby avoiding any chemical or physical modification of the original biofluid prior to the analysis. To achieve this, we employed a specific one-dimensional photo-CIDNP NMR pulse sequence that yields background-free hyperpolarization NMR spectra featuring signal-enhanced metabolite resonances exclusively within a few seconds of measurement time. Furthermore, the substantial analytical potential of the method is highlighted given that, out of a reservoir of a myriad of different small-molecule components, only a specific subset of clinically relevant targets, i.e., Trp, Tyr, His, Met, and, most interestingly, several additional metabolites, can be detected straightforwardly in their native environment. In addition, we explain how the method can be easily extended to allow for the high-throughput analysis of a large, metabolomically relevant number of samples, thereby underscoring the significant analytical capabilities of the approach.

## Experimental Section

### Sample Preparation

**Mixture 1** (pH 7.2) was prepared via adding 200 μL of stock solution 1 (Table S1), 60 μL of a 2 mM FMN stock solution, and 340 μL of D_2_O. For the preparation of urine samples, three different specimens from healthy volunteers were utilized (urine a, b, and c). One mL of each sample was lyophilized and, subsequently, reconstituted in the same volume of D_2_O. The sample of “**spiked urine 1**” (pH 6.8) comprised 300 μL of reconstituted urine a mixed with 1 μL of l-Trp (26.0 mM), 1 μL of l-His (204.4 mM), 1 μL of l-Tyr (6.6 mM), 1 μL of l-Met (210.0 mM), 60 μL of FMN stock solution (2 mM), and 236 μL of D_2_O, respectively. **Urine 2** and **urine 3** samples (pH 6.8) comprised 300 μL of reconstituted urine b and c, respectively, mixed with 60 μL of the FMN stock solution (2 mM) and 240 μL of D_2_O. For the preparation of serum samples, 2 mL of commercial human serum were lyophilized and, subsequently, reconstituted in D_2_O using the same volume of solvent. The “**spiked serum**” sample (pH 7.2) comprised 500 μL of reconstituted serum mixed with 12 μL of l-Trp (26.0 mM), 1 μL of l-His (204.4 mM), 2 μL of l-Tyr (6.6 mM), 1 μL of l-Met (210.0 mM), 60 μL of the FMN stock solution (2 mM), and 24 μL of D_2_O, respectively. The unmodified **serum** sample (pH 7.2) comprised 300 μL of reconstituted serum mixed with 60 μL of the FMN stock solution (2 mM) and 240 μL of D_2_O.

Samples and data from patients included in this study were provided by the Biobank Biobanco Hospital Universitario de La Princesa (ISCIII B.0000763) and were processed following standard operating procedures with the appropriate approval of the Ethics and Scientific Committees. Experimental procedures performed on human samples were approved by the Ethics Committee on Animal and Human Experimentation of the Universitat Autònoma de Barcelona (Approval number: CEEAH 5940).

### NMR Spectroscopy

NMR experiments were conducted using a Bruker Avance 600 MHz NMR spectrometer operating at a proton (^1^H) frequency of 600.13 MHz equipped with a triple-resonance Bruker TXI 5 mm ^1^H{^13^C/^15^N} room-temperature probe featuring a pulsed field gradient coil acting on the *z*-axis (Bruker Biospin, Rheinstetten, Germany). The probe temperature was kept at 298.0 K for all experiments. The data were acquired, processed, and analyzed using TopSpin 3.6.3 (Bruker Biospin, Rheinstetten, Germany). Prior to performing NMR experiments, all samples were subjected to external chemical shift referencing using a glass capillary insert containing TSP (10 mM in D_2_O) for axis calibration.

Throughout the entire study, a continuous wave (CW) diode laser (Cobolt 06-MLD, HÜBNER Photonics GmbH, Kassel, Germany) operated at a wavelength (λ) of 445 nm (max. nominal output power: 400 mW) equipped with appropriate fiber coupling optics was used as a light source. The laser light was guided into the NMR tube using an FP1000URT optical fiber (core diameter: 1 mm; Thorlabs Inc., Newton, NJ). The bare end of the optical fiber was placed inside the NMR tube via a coaxial insert (WGS-5-BL, SP Wilmad LabGlass, Vineland, NJ), ca. 1 mm above the active coil region of the probe. A coupling efficiency of approximately 65% was achieved using this setup. The laser itself was triggered by using a 20 ms voltage-gated pulse coming from a spare TTL line on the NMR spectrometer console.

For the collection of all hyperpolarization NMR data described here, a specific one-dimensional ^1^H photo-CIDNP NMR pulse sequence developed by Hore and co-workers was used which yields a “pure” photo-CIDNP spectrum and, thus, renders the subsequent subtraction of photo-CIDNP “light” and “dark” spectra superfluous.^16^ The experiment combines presaturation of (thermal) background magnetization by a string of composite π/2 pulses, each followed by a defocusing field gradient, and subsequent gated illumination during a grid of π pulses with a prescribed timing. This permits the acquisition of photo-CIDNP NMR spectra that are free from background magnetization, thereby avoiding the sensitivity loss and subtraction artifacts associated with difference spectroscopy (see text). All ^1^H photo-CIDNP NMR data were recorded in the time domain as free induction decays (FID; digital resolution: 64k) across a spectral width of 15.02 ppm (9014 Hz) as the sum of 16 transients using a recycle delay (d1) of 3 s between scans. FIDs were apodized applying an exponential function (0.2 Hz linebroadening) prior to Fourier transform (FT). Subsequently, the spectra were manually phased and baseline corrected. In all cases, a thermal 1D pulse-acquire ^1^H NMR spectrum was recorded and processed using identical acquisition and processing parameters, respectively, prior to performing the photo-CIDNP experiment. NMR signals were integrated using TopSpin 3.6.3 (Bruker Biospin, Rheinstetten, Germany). Deconvolution of NMR signals (when indicated) was performed using MestreNova 14.2.3 (Mestrelab Research, Santiago de Compostela, Spain). For further experimental details, refer to the Supporting Information.

## Results and Discussion

In order to test the feasibility of performing photo-CIDNP experiments on unmodified complex fluids of biological origin, we decided to pursue a sequential experimental strategy: (i) Prior to performing photo-CIDNP NMR studies of untreated human urine and serum samples, the feasibility of recording photo-CIDNP NMR spectra of multiple molecules present in a molecularly congested environment using our setup was explored. To achieve this, we carried out the experiment using an aqueous complex mixture of the 20 naturally occurring L-amino acids. (ii) In a second step, the same experiment was conducted on a sample of normal human urine enriched with the four amino acids known to be polarizable via the photo-CIDNP effect, i.e., Tyr, Trp, His, and Met. (iii) Third, the acquisition of a 1D photo-CIDNP NMR spectrum of a completely untreated sample of normal human urine was performed. (iv) Finally, steps ii and iii were repeated examining amino acid-doped and pristine samples of human serum, respectively (c.f. Supporting Information, Scheme S1).

In a first step, thermal and photo-CIDNP ^1^H NMR experiments were conducted on aqueous mixture 1, which contained the 20 naturally occurring amino acids in low, physiologically representative concentrations and a small amount of the photosensitizer flavin mononucleotide (FMN; *c* = 0.2 mM), which is essential for observing the biomolecular photo-CIDNP effect.

**Figure 1:**
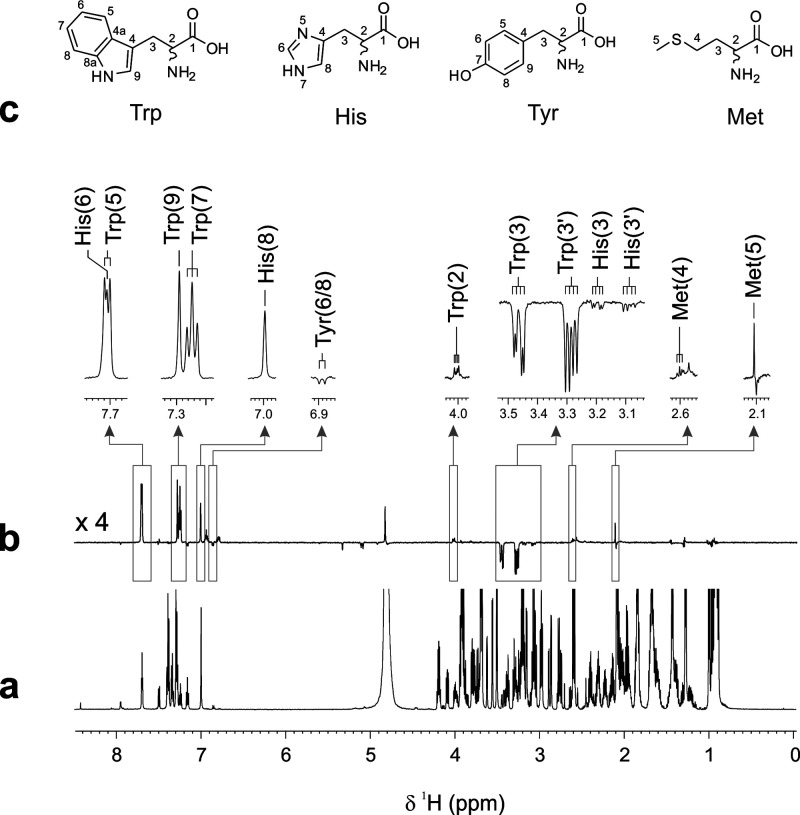
Comparison of one-dimensional (a) ^1^H and (b) ^1^H photo-CIDNP NMR spectra of “mixture 1” (pH 7.2; *T* = 298 K). In the upper part, expanded aliphatic and aromatic regions of the ^1^H photo-CIDNP spectrum are shown together with the respective resonance assignments. (c) Molecular structures of tryptophan, histidine, tyrosine, and methionine together with the respective numbering scheme used for all ^1^H nuclei. Photo-CIDNP NMR spectra were acquired with 16 scans, a recycle delay (d1) of 3 s, and a laser illumination time of 350 ms per scan using a state-of-the-art continuous wave diode laser (max. nominal output power: 400 mW) emitting at a wavelength (λ) of 445 nm (cf. Supporting Information).

Figure 1 compares the thermal ^1^H NMR spectrum of mixture 1 (Figure 1a) with the photo-CIDNP NMR spectrum of the same sample (Figure 1b), exclusively highlighting hyperpolarized amino acid nuclei resonances. While the thermal ^1^H NMR spectrum of mixture 1 (Figure 1a) shows a multitude of partially overlapping NMR signals, the acquisition of the photo-CIDNP NMR data of the same sample yields a clean, background-free spectral output showing only the hyperpolarized signals of the four photo-CIDNP-active amino acids (Figure 1b). In particular, protons H2, H5, H7, and H9 of tryptophan show absorptive polarization, whereas the two β-CH_2_ nuclei, H3 and H3′, are emissively polarized. In the case of histidine, the aromatic ring protons H6 and H8 exhibit absorptive polarization while the polarization signals representing the β-CH_2_ protons are weakly emissive. Furthermore, absorptive polarization for the H4 and H5 protons of the aliphatic amino acid methionine is observed. In the case of tyrosine—present in a much lower concentration in mixture 1 than the other three photo-CIDNP-active amino acids—only the emissively polarized H6,8 protons can be detected. This observation is consistent with the polarization pattern found in ^1^H photo-CIDNP NMR spectra of isolated tyrosine, typically characterized by a strong emissive polarization for the aromatic ring protons H6,8 and only relatively weak absorptive polarization signals for the aromatic H5,9 and the β-CH_2_ nuclei.

Hence, we were able to hyperpolarize simultaneously Tyr, Trp, His, and Met within a relatively complex aqueous mixture containing numerous other, nonpolarizable metabolites employing our photo-CIDNP NMR setup. Moreover, the polarization pattern of the individual amino acid resonances as well as their sign, i.e., emissive or absorptive, and the relative signal intensities resemble those found in photo-CIDNP NMR spectra of the isolated aromatic amino acids recorded in the absence of cosolutes.^12,17^ Interestingly, all four photo-CIDNP active amino acids were hyperpolarized simultaneously, although other structurally similar molecules were present in the mixture. In principle, these could also have interacted with the FMN photosensitizer thereby preventing or attenuating the generation of photo-CIDNP. As such, this preliminary result gave reason to believe that hyperpolarization of the four photo-CIDNP-active amino acids might also be observable in more complex, metabolomically relevant, environments.

**Figure 2:**
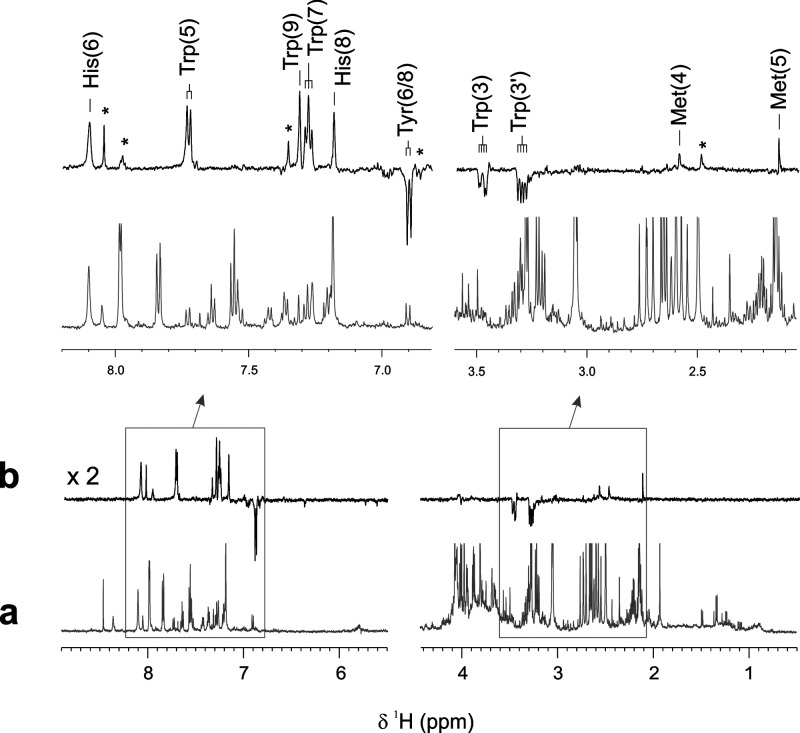
Comparison of one-dimensional (a) ^1^H and (b) ^1^H photo-CIDNP NMR spectra of a sample of normal human urine (pH 6.8) spiked with the four photo-CIDNP-active amino acids (“spiked urine 1”: c (Trp): 0.043 mM; c (His): 0.341 mM; c (Tyr): 0.011 mM; c (Met): 0.350 mM; c (FMN): 0.2 mM; pH 6.8; *T* = 298 K). In the upper part, expanded aliphatic and aromatic regions of both spectra are shown, together with the respective resonance assignments. Unassigned hyperpolarized resonances are marked with an asterisk.

In a subsequent step, both thermal and ^1^H photo-CIDNP NMR spectra of a sample of normal human urine (pH 6.8) spiked with the four photo-CIDNP active amino acids (“spiked urine 1”) were recorded (Figure 2). As expected, the thermal ^1^H NMR spectrum of this mixture (Figure 2a) is characterized by the presence of a large number of NMR signals of very different intensities thereby reflecting the wide range of metabolite concentrations found in normal human urine. Furthermore, many of these signals show considerable overlap which renders the detection and identification of poorly concentrated metabolites difficult. In contrast, the photo-CIDNP spectrum of the same sample (Figure 2b) shows numerous hyperpolarized NMR resonances, while all other NMR signals present in the thermal spectrum are absent. A straightforward analysis allowed the identification of most of these hyperpolarized NMR resonances by comparison of the respective chemical shifts with reported literature values and/or their characteristic polarization signal pattern. In particular, polarized signals were attributed to ^1^H nuclei of either histidine (polarized ^1^H nuclei: H6, H8), tryptophan (H2, H3, H3′, H5, H7, and H9), tyrosine (H6,8), or methionine (H4, H5). While hyperpolarization signals representing the aliphatic beta-protons of tyrosine and histidine are absent in this spectrum, several additional polarization signals of unknown origin, marked with an asterisk in Figure 2b, can clearly be observed.

Thus, the analysis of these data shows that hyperpolarization of the four amino acids Tyr, Trp, His, and Met—together with several other unknown urine metabolites (see below)—via the photo-CIDNP effect can be detected in a complex biofluid, e.g., human urine, and that its complex biological matrix does not hinder the hyperpolarization of these metabolites. This finding is anything but trivial given that, for example, the specific and nonspecific binding of the low-concentration photosensitizer (FMN; *c* = 0.2 mM) to other, photo-CIDNP-inactive molecular, ionic and/or, complexing components as well as the competition for triplet-excited FMN species between photo-CIDNP substrates and other molecular species found in human urine^18^ could, in principle, have prevented the detection of polarization signals entirely.^17,19^

**Figure 3:**
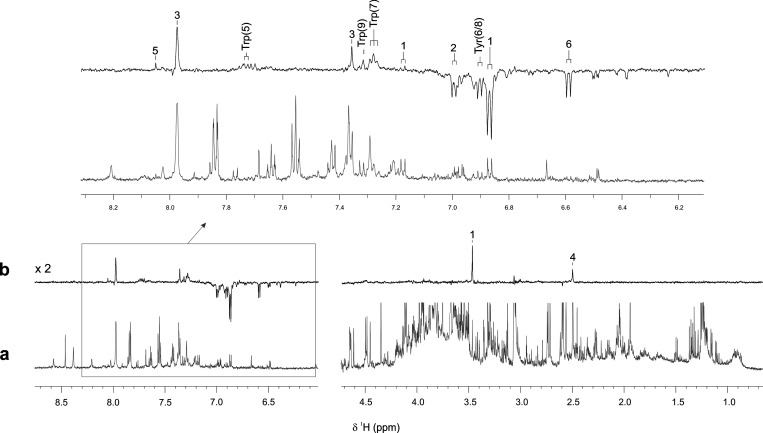
Comparison of one-dimensional (a) ^1^H and (b) ^1^H photo-CIDNP NMR spectra of an untreated sample of normal human urine, “urine 2” (pH 6.8; *T* = 298 K). In the upper part, an expanded section of the aromatic region of both spectra is highlighted. Assignments for hyperpolarized resonances of Tyr, Trp, 4-hydroxyphenylacetic acid (1), 4-hydroxybenzoic acid (2), urocanic acid (3), homocysteine (4), guanosine (5), and 6-hydroxynicotinic acid (6) are indicated.

Encouraged by these results, we then recorded both thermal and photo-CIDNP ^1^H NMR spectra of a completely untreated sample of human urine (“urine 2”) in the presence of the photosensitizer FMN (*c* = 0.2 mM) (Figure 3). A first analysis of the data shows that the photo-CIDNP ^1^H NMR spectrum of the pristine urine sample (Figure 3b) yields a much cleaner, i.e. background-free, result as compared to its thermal counterpart (Figure 3a). Moreover, it is significantly different from the hyperpolarized NMR spectra obtained for both “mixture 1” and “spiked urine 1” (Figure 1b and Figure 2b). Whereas the aliphatic part of the photo-CIDNP spectrum shown in Figure 3b exhibits only two absorptive signals, its aromatic region features a relevant number of hyperpolarized NMR resonances whose chemical shift positions differ from those assigned in Figure 1b and Figure 2b, respectively (see below). In addition, hyperpolarized resonances of the aromatic amino acids tyrosine (H6,8) and tryptophan (H5, H7, H9) are also detectable (Figure 3b). In contrast, signals of histidine and methionine are completely absent in the spectrum. This finding can most likely be attributed to a lower concentration of these species in the sample. Also, the absence of these signals might be due to the fact that, unlike Tyr and Trp, both His and Met compete less favorably for the amount of triplet-excited flavin molecules present in solution after each laser flash.^17^ This, in turn, leads to a less pronounced photo-CIDNP effect in the resulting spectrum.

A more detailed analysis of the hyperpolarization spectrum shown in Figure 3b identifies a number of emissive doublet signals located both “upfield” and “downfield” of the emissive resonance representing the H6,8 protons of tyrosine, as well as several absorptive singlets found in both the aromatic and the aliphatic regions of the spectrum. Interestingly, many of these NMR signals can barely be observed in the thermal ^1^H NMR spectrum of the sample. Applying state-of-the-art NMR resonance identification methods based on the analysis and comparison of chemical shift information^20^ in combination with complementary NMR data (see Supporting Information: Materials and Methods; Figures S1 and S2), we were able to assign these resonances unambiguously and identify a total of six additional clinically relevant urinary metabolites (Figure 3b, 1–6): 4-hydroxyphenylacetic acid (1), a tyrosine metabolite, acts as a biological molecular marker for certain diseases. For example, its upregulation in neonatal urine appears to be indicative of type II/III tyrosinemia.^21^ 4-hydroxybenzoic acid (2), another para-hydroxy-substituted aromatic metabolite, is related to ubiquinone biosynthesis and has been described as a urinary biomarker for the detection of colorectal cancer.^22^ Both of these molecules show a very similar photo-CIDNP hyperpolarization pattern compared to tyrosine. In addition, we unequivocally assigned the remaining photo-CIDNP signals to the metabolites urocanic acid (3), a derivative of the amino acid histidine, homocysteine (4), involved in the metabolism of methionine, guanosine (5),^23^ and 6-hydroxynicotinic acid (6). Abnormal levels of these metabolites found in various human body fluids are also indicative of a number of medical conditions.^24,25^ Interestingly, it becomes evident that some of these additionally identified metabolites, i.e., hydroxyphenylacetic acid, 4-hydroxybenzoic acid, urocanic acid, and homocysteine, are also present in the photo-CIDNP spectrum of the sample “spiked urine 1” (Figure 2; resonances marked with asterisks).

Next, we investigated whether the photo-CIDNP technique can also be applied to analytes present in metabolomically relevant matrices other than urine. Thus, the observation of photo-CIDNP in both amino acid-doped and fully untreated samples of normal human serum^26^ following identical procedures as described before was explored (c.f. Supporting Information). With regard to performing NMR experiments requiring sample illumination, we encountered that undiluted human serum is a rather challenging medium given that it is often, unlike urine, not fully transparent but rather turbid (serum turbidity is usually caused by cryoprecipitation of lipid components during freezing and thawing cycles), resulting in a relatively high optical density of the sample solution. Accordingly, the laser light exiting the tip of the optical fiber does not penetrate the entire active volume of the NMR sample, thereby producing less triplet-excited photosensitizer molecules upon illumination. Thus, we expected that the photo-CIDNP effect should be less pronounced as compared to the above-mentioned results achieved with human urine samples.

**Figure 4:**
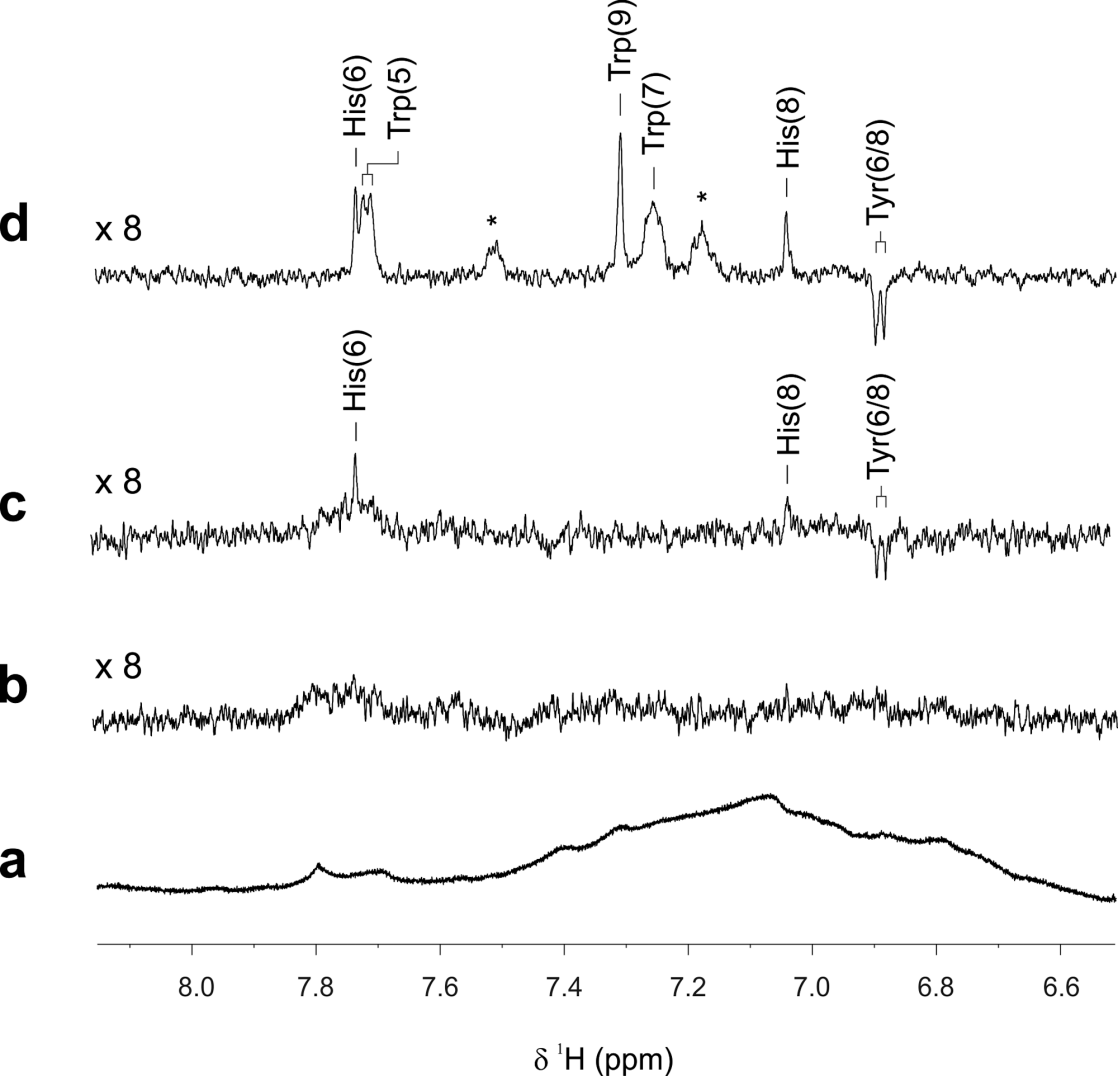
Comparison of the aromatic regions of (a) ^1^H, (b) ^1^H CPMG, and (c) ^1^H photo-CIDNP NMR spectra of a pristine sample of normal human serum (pH 7.2; *T* = 298 K). In addition, the aromatic region of (d) a ^1^H photo-CIDNP NMR spectrum of an amino acid-doped serum sample is shown. Resonance assignments of hyperpolarized signals found in the photo-CIDNP spectra are indicated. Unidentified polarization signals are marked with an asterisk.

Figure 4 compares the aromatic regions of the photo-CIDNP spectra of unmodified (Figure 4c) human serum (“serum”) and the amino acid-doped serum sample (“spiked serum”: c (Trp): 0.520 mM; c (His): 0.341 mM; c (Tyr): 0.022 mM; c (Met): 0.350 mM; c (FMN): 0.2 mM; Figure 4d). In addition, 1D ^1^H (Figure 4a) and ^1^H Carr–Purcell–Meiboom–Gill (CPMG) (Figure 4b) NMR spectra of the unmodified serum sample acquired prior to conducting the photo-CIDNP experiment are shown for comparison.^27^ While the aromatic region of the ^1^H NMR spectrum of untreated human serum is characterized by broad signals originating from high-molecular-weight components, e.g., proteins and lipids, typically found in this medium, acquisition of a state-of-the-art ^1^H CPMG experiment yields an aromatic spectral region void of any signals. Interestingly, both photo-CIDNP spectra shown in Figure 4c,d, respectively, do clearly feature polarization-enhanced NMR signals in the aromatic region, albeit smaller than those detected in samples of human urine, corresponding to photo-CIDNP-active amino acids. In particular, the polarization spectrum representing the amino acid-doped sample exhibits significantly broadened photo-CIDNP signals for Tyr, His, and Trp. However, no signals can be observed for the amino acid methionine. This observation can possibly be attributed to a less favorable competition of Met for triplet-excited FMN molecules.^17^ In addition, two unassigned hyperpolarized NMR signals, indicated with asterisks in Figure 4d, were also detected. Interestingly, their chemical shift positions coincide with those of the H6 and H8 protons of tryptophan, previously unknown to exhibit the photo-CIDNP effect (Figure S3).

With regard to the unaltered serum sample, weakly hyperpolarized His (H6 and H8) and Tyr (H6/8) resonances were detected. Importantly, these signals are entirely absent in the ^1^H CPMG spectrum (Figure 4b) acquired prior to conducting the photo-CIDNP experiment. Furthermore, polarization signals stemming from other metabolites, e.g., Trp and Met, cannot be observed in the spectrum. This finding is most likely due to a substantially lower concentration of these species in this sample of human serum.

Subsequently, the signal enhancement of the hyperpolarized ^1^H nuclei of the analyzed samples was measured (Supporting Information, Table S2). ^1^H photo-CIDNP signal enhancement factors of up to 6.8-fold were obtained from this analysis. These results were consistent with values reported in studies of other types of biological analytes under similar standard experimental photo-CIDNP conditions.^12,17^ However, it cannot be excluded that the complex matrix of the biofluid has an influence on the extent of photo-CIDNP enhancement. This should be further investigated in a specific study. In addition, further development and improvement of the method could lead to a significant increase in the signal gain (see Conclusions and Outlook).

In a final step, a preliminary evaluation of the reproducibility of the method was carried out. For this purpose, an unmodified human urine sample (“urine 3”) was analyzed according to the described method (Scheme S1). The evaluation was based on the results of three replicates obtained from three independent sample preparations starting from the same urine stock solution. The coefficients of variation (CV) associated with the absolute integral values of the NMR peaks range from 2 to 4%, which means that variations in metabolite concentrations of above 4% can be detected with the described method. This value can be even lower when working with normalized peak integrals. The results obtained show a high degree of reproducibility, which compares very favorably with other hyperpolarization methods.^28^ This high reproducibility of the photo-CIDNP NMR method is not surprising, given the minimal sources of variability associated with the experimental setup (Scheme S1).

## Conclusions and Outlook

Here, we have demonstrated that the acquisition of 1D ^1^H photo-CIDNP NMR data of unmodified samples of human urine and serum is feasible; can be accomplished in a minimally invasive manner, i.e., hyperpolarization is generated within the analyte molecules in their native biological matrix; and yields a clean, background-free spectral output featuring photo-CIDNP-enhanced NMR signals of the amino acids tyrosine, tryptophan, histidine, and methionine. Interestingly, our study also showed that other metabolites found in human biofluids can be hyperpolarized as well using this method. The specific detection and identification of these species as part of a metabolomic analysis is of importance given their high clinical significance for the diagnosis and treatment of different pathologies, e.g., Alzheimer’s disease,^29^ Lewy body dementia,^30^ Parkinson’s disease,^31^ and leukemia.^32^ Furthermore, the method is fast, robust, and highly reproducible. It is assumed that its application can be particularly interesting for the metabolomic analysis of samples characterized by an inherently low metabolite concentration, e.g., cerebrospinal fluid or tear fluid. Two additional characteristics of this analytical approach are of great importance in the context of analyses of biological samples as they facilitate the comparison and identification of metabolites with current spectral databases: (i) all hyperpolarized metabolite signals appear at identical chemical shift positions in the spectrum as compared to the original sample; (ii) although we did not add any type of standard buffer, e.g., phosphate, in this study, the photo-CIDNP method allows its use to control the pH of the medium.^17^ We believe that no other NMR hyperpolarization technique has, as of yet, achieved a similar level of compatibility with general analytical procedures for the study of biological samples. In addition, the method provides highly reproducible results due to minimal sources of variability related to the experimental setup. Accordingly, the work presented here opens a new branch of research in the field of hypersensitive NMR analyses of complex biological mixtures. In particular, it will enable the development of new applications in the field of metabolic profiling and metabolomic studies, as well as in other areas where complex mixtures containing clinically relevant metabolites need to be efficiently characterized. Due to its noninvasive character and the relatively modest setup requirements, a straightforward incorporation of the approach into existing NMR-based metabolomic workflows seems likely.

The photo-CIDNP method as applied to unmodified biofluids bears a significant analytical potential, and thus, numerous lines of investigation should be pursued in subsequent studies to improve its overall performance. First, a dedicated study to explore and optimize the sensitivity gain achieved with the photo-CIDNP method as applied to biofluids needs to be carried out in the future. Significant experimental and theoretical work into increasing the sensitivity of biological photo-CIDNP NMR measurements has been carried out in the last 15 years allowing the lowering of the detection limit down to the low nM concentration range.^14,15^ The implementation of many of these improvements for the photo-CIDNP-based analysis of metabolomically relevant biofluids must be investigated. Second, a class of polarizable molecular targets needs to be identified. In general, the photo-CIDNP effect is limited to molecules with low ionization energies, e.g., aromatics, able to participate in the radical pair mechanism (RPM) responsible for the generation of CIDNP.^17^ Hence, biological photo-CIDNP was known to be mainly restricted to the four amino acids tyrosine, tryptophan, histidine, and methionine. This work has shown that at least several additional metabolites can also be polarized via the photo-CIDNP effect. As such, it has to be explored whether other (aromatic) metabolites can be polarized as well. Given that a relevant number of newly identified photo-CIDNP-active small molecules has been reported very recently^33^ we believe that the approach might be applied as a more general metabolomics screening method in the future. In particular, its use might be of significant benefit for targeted analyses as the method’s substrate selectivity can be further increased by variation of the photo-CIDNP photosensitizer and/or the pH of the sample solution.^17^ Third, means to incorporate the method into metabolomic workflows need to be found and studies into the quantifiability of photo-CIDNP signals stemming from polarizable metabolite molecules present in complex biofluids have to be carried out (e.g., via exploring time-resolved photo-CIDNP experiments). This would extend the method to allow for a reliable, high-throughput screening of a metabolomically relevant number of samples. Finally, we believe that our approach can be merged with low-field detection methods (e.g., NMR benchtop solutions). The advantages of this will be twofold: (i) The photo-CIDNP effect is more pronounced at lower magnetic field strengths than used here. Hence, hyperpolarizing analytes at these lower field strengths should, in principle, yield higher amounts of nuclear polarization. (ii) The introduction of benchtop NMR solutions facilitates the use of (automated) flow-probe-assisted online reaction monitoring in situ. In addition, a fiberless “NMR torch” illumination protocol^34^ would be combinable with such an approach.

## Supporting Information Available

Detailed description of material and methods (chemicals and samples, sample preparation, NMR experiments and signal enhancement measurements); workflow scheme; thermal and photo-CIDNP spectra of “urine 2” sample; 1D ^1^H selective TOCSY of “urine 2” sample; thermal and photo-CIDNP spectra of “spiked serum” sample; evaluation of the reproducibility of the method; signal enhancement factors of hyperpolarized resonances in photo-CIDNP spectra (PDF)
