# Supplementary material for: Hyperpolarization-Enhanced NMR Spectroscopy of Unaltered Biofluids Using Photo-CIDNP
Source: Anal Chem. 2023 Dec 18;96(1):102–9. doi: 10.1021/acs.analchem.3c03215 (PMC10782414; doi:10.1021/acs.analchem.3c03215)
Supplement: Supplementary file 1 — ac3c03215_si_002.pdf [file ac3c03215_si_002.pdf]

Supporting Information:

Hyperpolarization-Enhanced NMR Spectroscopy of Unaltered Biofluids Using Photo-CIDNP

Lars T. Kuhn,[†,*] Stefan Weber,[†] Joachim Bargon,[‡] Teodor Parella,[§] and Míriam Pérez-Trujillo[§,*]

[†]Institut für Physikalische Chemie, Albert-Ludwigs-Universität Freiburg, Albertstr. 21, 79104 Freiburg i. Br., Germany; [‡]Institut für Physikalische und Theoretische Chemie, Rheinische Friedrich-Wilhelms-Universität Bonn, Wegelerstr. 12, 53115 Bonn, Germany; [§]Servei de Ressonància Magnètica Nuclear, Facultat de Ciències i Biociències, Universitat Autònoma de Barcelona, 08193 Cerdanyola del Vallès, Catalonia, Spain

Table of Contents

Materials and Methods S2

Chemicals and samples S2

Sample preparation S2

NMR experiments S3

Signal enhancement measurement S3

Results and Discussion S5

Workflow: NMR analysis of unaltered biofluids via photo-CIDNP S5

Thermal and photo-CIDNP spectra of the “urine 2” sample S6

1D ^{1}H selective TOCSY experiments of the “urine 2” sample S7

Thermal and photo-CIDNP spectra of the “spiked serum” sample S8

Evaluation of the reproducibility of the method S9

Signal enhancement factors (ε) of hyperpolarized resonances in photo-CIDNP spectra S10

[*]To whom correspondence should be addressed: lars.kuhn@physchem.uni-freiburg.de; miriam.perez@uab.cat

Materials and Methods

Chemicals and samples

L-amino acids, 3-(trimethylsilyl)-[2,2,3,3-$^{2}H_4$]-propionic acid sodium salt (TSP), flavin mononucleotide (FMN) and human serum were purchased from Sigma-Aldrich S.A. (Madrid, Spain). Deuterium oxide (99.96% D) was obtained from CortecNet SAS (Voisins-le-Bretonneux, France). Samples and data from patients included in this study were provided by the Biobank Biobanco Hospital Universitario de La Princesa (ISCIII B.0000763) and they were processed following standard operating procedures with the appropriate approval of the Ethics and Scientific Committees. A statement declaring the consent of all participating subjects was obtained. Experimental procedures performed on human samples were approved by the Ethics Committee on Animal and Human Experimentation of the Universitat Autònoma de Barcelona (Approval number: CEEAH 5940).

Sample preparation

Individual L-amino acid stock solutions were prepared in D_2O (1 mL each) using concentrations indicated in Table S-1. Stock solution 1 (2.0 mL, 129.03 mM) was made by mixing indicated amounts of individual amino acid stock solutions (volumes indicated in the table). In addition, a 2 mM stock solution of the photo-CIDNP dye flavin mononucleotide (FMN) was prepared in D_2O. **Mixture 1** (pH 7.2) was prepared via adding 200 µL of stock solution 1, 60 µL of the FMN stock solution and 340 µL of D_2O.

For the preparation of urine samples, three different specimens from healthy volunteers were utilized (urine a, urine b and urine c). 1 mL of each sample was lyophilized and, subsequently, reconstituted in the same volume of D_2O. The sample of "**spiked urine 1"** (pH 6.8) comprised 300 µL of reconstituted urine a mixed with 1 µL L-Trp (26.0 mM), 1 µL L-His (204.4 mM), 1 µL L-Tyr (6.6 mM), 1 µL L-Met (210.0 mM), 60 µL of FMN stock solution (2 mM), and 236 µL of D_2O, respectively. **Urine 2** and **urine 3** samples (pH 6.8) comprised 300 µL of reconstituted urine b and c, respectively, mixed with 60 µL of the FMN stock solution (2 mM) and 240 µL of D_2O.

For the preparation of serum samples, 2 mL of commercial human serum were lyophilized and, subsequently, reconstituted in D_2O using the same volume of solvent. The **"spiked serum"** sample (pH 7.2) comprised 500 µL of reconstituted serum mixed with 12 µL L-Trp (26.0 mM), 1 µL L-His (204.4 mM), 2 µL L-Tyr (6.6 mM), 1 µL L-Met (210.0 mM), 60 µL of the FMN stock solution (2 mM) and 24 µL of D_2O, respectively. The unmodified **serum** sample (pH 7.2) comprised 300 µL of reconstituted serum mixed with 60 µL of the FMN stock solution (2 mM) and 240 µL of D_2O.

Table S-1. Concentrations for amino acid components found in **mixture 1** together with the concentrations of all stock solutions used for its preparation.

Amino acid (symbol)		**Amino acid individual stock solution** [(a)]	**Stock solution1** (2,00 mL; 129,03 mM)[(b)]		**Mixture 1** (0,6mL; 43,01mM)[(c)]
		Concentration (mM)	Volume of stock sol. (µL)	Concentration (mM)	Concentration (mM)
Alanine-L	Ala	220,1	70	7,70	2,57
Arginine-L	Arg	207,3	95	9,85	3,28
Asparagine-L	Asn	220,4	70	7,71	2,57
Aspartic acid-L	Asp	30,1	135	2,03	0,68
Cysteine-L	Cys	29,7	140	2,08	0,69
Glutamic Acid-L	Glu	30,6	130	1,99	0,66
Glutamine-L	Gln	234,1	45	5,27	1,76
Glycine	Gly	12,0	120	0,72	0,24
Histidine-L	His	204,4	145	14,82	4,94
Isoleucine-L	Ile	257,8	75	9,67	3,22
Leucine-L	Leu	197,6	60	5,93	1,98
Lysine-L, HCl	Lys	199,2	75	7,47	2,49
Methionine-L	Met	210,0	95	9,97	3,32
Phenylalanine-L	Phe	173,9	70	6,08	2,03
Proline-L	Pro	246,0	60	7,38	2,46
Serine-L	Ser	227,5	65	7,39	2,46
Threonine-L	Thr	196,5	120	11,79	3,93
Tryptophan-L	Trp	26,0	235	3,05	1,02
Tyrosine-L	Tyr	6,6	120	0,40	0,13
Valine-L	Val	205,8	75	7,72	2,57
TOTAL	-	-	2000	129,03	43,01

(a) 1 mL of a stock solution of each amino acid was prepared using concentrations indicated in the table. (b) Stock solution 1 (2,0 mL; 129,03 mM): indicated amounts of amino acid stock solutions were mixed to obtain stock solution 1. (c) **Mixture 1** (0,6 mL; 43,21 mM): 200 μL of stock solution 1 were mixed with 60 μL of the FMN stock solution (2mM) and 340 μL of D_2O.

NMR experiments

NMR experiments were conducted using a Bruker Avance 600 MHz NMR spectrometer operating at a proton (^1H) frequency of 600.13 MHz equipped with a triple-resonance Bruker TXI 5 mm $^1H\{^{13}C/^{15}N\}$ room-temperature probe featuring a pulsed field gradient coil acting on the *z*-axis (Bruker Biospin, Rheinstetten, Germany). The probe temperature was kept at 298.0 K for all experiments. The data were acquired, processed, and analyzed using TopSpin 3.6.3 (Bruker Biospin, Rheinstetten, Germany). Prior to performing NMR experiments, all samples were subjected to external chemical shift referencing using a glass capillary insert containing TSP (10 mM in D_2O) for axis calibration.

Throughout the entire study, a continuous wave (CW) diode laser (Cobolt 06-MLD, HÜBNER Photonics GmbH, Kassel, Germany) operating at a wavelength (λ) of 445 nm (max. nominal output power: 400 mW) equipped with appropriate fiber coupling optics was used as a light source. The laser light was guided into the NMR tube using an FP1000URT optical fiber (core diameter: 1 mm; Thorlabs Inc., Newton, NJ). The bare end of the optical fiber was placed inside the NMR tube via a coaxial insert (WGS-5-BL, SP Wilmad LabGlass, Vineland, NJ), ca. 1 mm above the active coil region of the probe. A coupling efficiency of approximately 65% was achieved using this setup. The laser itself was triggered using a 20 ms voltage-gated pulse coming from a spare TTL line on the NMR spectrometer console.

For the collection of all hyperpolarization NMR data described here, a specific one-dimensional ^1H photo-CIDNP NMR pulse sequence developed by Hore and co-workers was used which yields a “pure” photo-CIDNP spectrum and, thus, renders the subsequent subtraction of photo-CIDNP “light” and “dark” spectra superfluous (M. Goez, K. H. Mok, P. J. Hore, *J. Magn. Reson.* **2005**, 177, 236-246). The experiment combines presaturation of (thermal) background magnetization by a string of composite π/2 pulses, each followed by a defocusing field gradient, and subsequent gated illumination during a grid of π pulses with a prescribed timing. This permits the acquisition of photo-CIDNP NMR spectra that are free from background magnetization thereby avoiding the sensitivity loss and subtraction artefacts associated with difference spectroscopy (see text). All ^1H photo-CIDNP NMR data were recorded in the time domain as free induction decays (FID; digital resolution: 64k) across a spectral width of 15.02 ppm (9014 Hz) as the sum of 16 transients using a recycle delay (d1) of 3 s between scans. FIDs were apodised applying an exponential function (0.2 Hz linebroadening) prior to Fourier transform (FT). Subsequently, the spectra were manually phased and baseline corrected. In all cases, a thermal 1D pulse-acquire ^1H NMR spectrum was recorded and processed using identical acquisition and processing parameters, respectively, prior to performing the photo-CIDNP experiment. NMR signals were integrated using TopSpin 3.6.3 (Bruker Biospin, Rheinstetten, Germany). Deconvolution of NMR signals (when indicated) was performed using MestreNova 14.2.3 (Mestrelab Research, Santiago de Compostela, Spain).

In case of mixture 1 and human urine samples, a standard 1D ^1H NMR experiment was conducted to obtain a (thermal) reference spectrum. A water-suppressed 1D ^1H Carr-Purcell-Meiboom-Gill (CPMG) NMR spectrum with T_2-filter was acquired as thermal reference for human serum samples. In both cases, identical standard acquisition and processing parameters as compared to the photo-CIDNP experiment were used. In addition, standard 1D ^1H selective TOCSY experiments were acquired for the sample “urine 2”. Irradiated frequencies were 7.202, 7.174, 6.902, 6.867, 6.995, and 7.769 ppm, respectively. TOCSY spectra were acquired as the sum of 1024 transients using a mixing time of 60 ms, a recycle delay of 1 s, and a spectral width of 20.02 ppm (12020 Hz). Prior to Fourier transform, FIDs were apodised using an exponential function (0.6 Hz linebroadening) and the resulting spectra were manually phased and baseline corrected.

Spectral NMR signal assignments were carried out following standard operating procedures. In particular, the HMDB (Human Metabolome Database) spectral data base (D. S. Wishart, A. Guo, E. Oler, *et al.*, *Nucl. Acids Res*. **2021**, *50*, D622) was utilized to assign NMR signals to their corresponding metabolites. In addition, the comparison of the photo-CIDNP pattern of polarized signals with previously reported data (L. T. Kuhn, *Top. Curr. Chem*. **2013**, *338*, 229; P. J. Hore, R. W. Broadhurst, *Prog. Nucl. Magn. Reson. Spectrosc*. **1993**, *25*, 345; S. Stob, R. M. Scheek, R. Kaptein, *Photochem. Photobiol*. **1989**, *49*, 717) as well as the analysis of selective ^1H-^1H TOCSY (TOtal Correlation SpectroscopY) data helped to confirm such assignments.

Signal enhancement measurement

Photo-CIDNP signal enhancement factors (ε) were calculated according to Equation 1 (J. E. Bramham, A. P. Golovanov, *Commun. Chem.* **2022**, *5*, 90):

$$\varepsilon = \frac{|I_\mathrm{L}|}{I_\mathrm{D}} \qquad \text{(Equation 1)}$$

where I_L and I_D represent the integral of the respective NMR signal in "light" and "dark" spectra, respectively. In our case, we utilized a modified version of 'Equation 1' taking into account that the photo-CIDNP pulse sequence used in this study yields "pure" hyperpolarization spectra void of any thermal background magnetization. Accordingly, 'Equation 1' was modified to yield 'Equation 2', which was used here for the calculation of signal enhancement factors:

$$\varepsilon = \frac{|I_{\mathrm{CIDNP}}| + I_{\mathrm{T}}}{I_{\mathrm{T}}} \qquad \text{(Equation 2)}$$

where I_{CIDNP} and I_T are the integrals of the signal in question both in the photo-CIDNP (I_{CIDNP}) and in the thermal (I_T) spectrum, respectively, which was acquired and processed using identical acquisition and processing parameters.

Results and Discussion

Workflow: NMR analysis of unaltered biofluids via photo-CIDNP

Scheme S-1. Schematic representation of the workflow applied to biofluids for their NMR analysis via photo-chemically induced dynamic nuclear polarization (photo-CIDNP)

1. Transfer of the biofluid into a standard Eppendorf tube (e.g. 570 µL).

↓

2. Addition of the FMN photosensitizer stock solution to the Eppendorf tube to yield a final FMN concentration of 0.2 mM (final sample volume: 600 µL).

↓

3. Thorough mixing of the sample solution using a vortex mixer followed by centrifugation.

↓

4. Transfer of the sample solution into a 5 mm NMR standard tube.

↓

5. Connection of the NMR tube with the optical insert carrying the optical fibre which, in turn, is coupled with the light source, e.g., laser.

↓

6. Transfer of the NMR tube into the NMR spectrometer.

↓

7. Acquisition of the photo-CIDNP NMR spectrum.

↓

8. Standard NMR processing & analysis.

Thermal and photo-CIDNP spectra of the "urine 2" sample

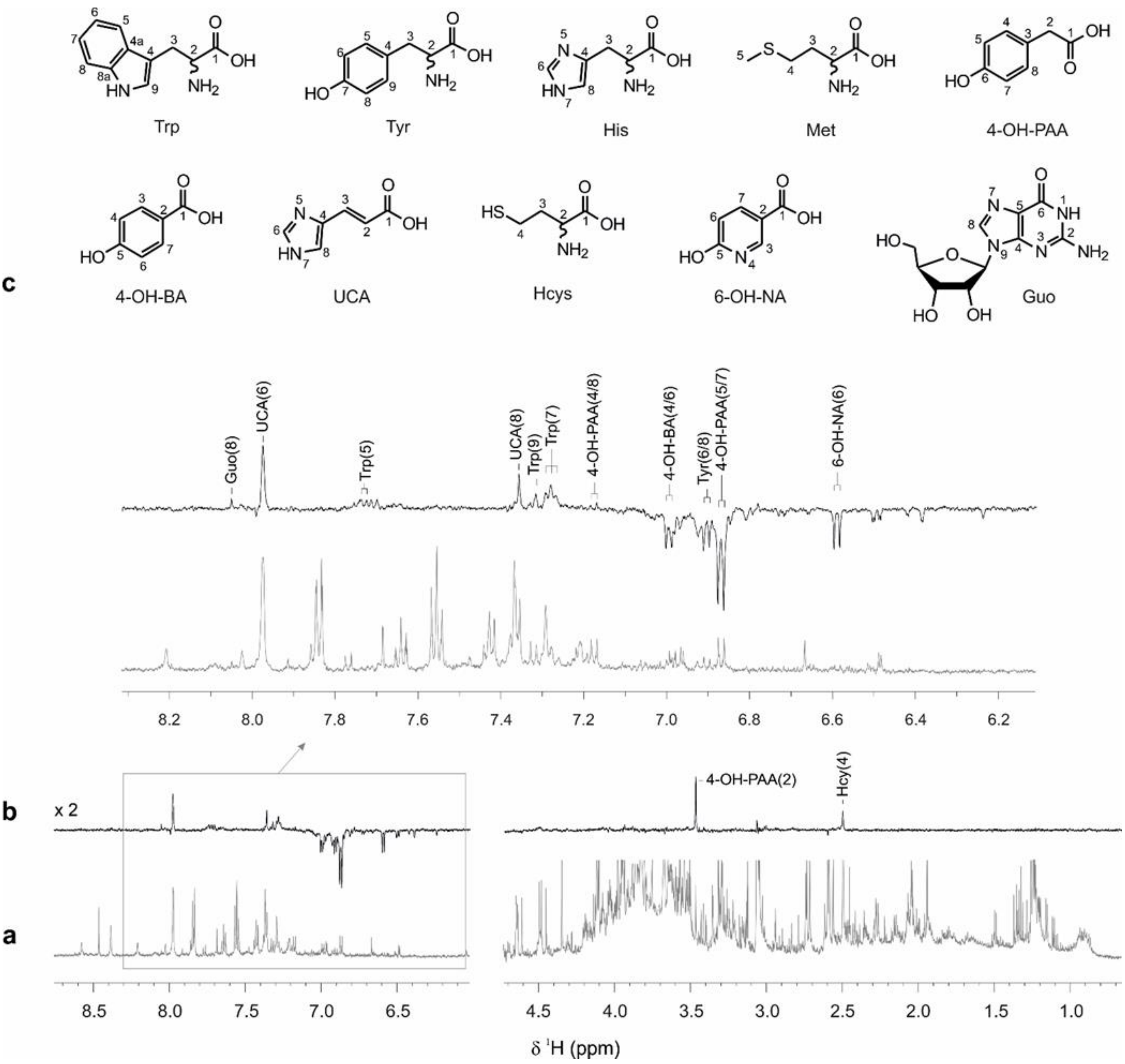

Figure S-1. Comparison of one-dimensional (a) ^{1}H and (b) ^{1}H photo-CIDNP NMR spectra of an untreated sample of normal human urine, "**urine 2**" (pH 6.8). In the upper part, an expanded section of the aromatic region of both spectra is highlighted. Assignments for hyperpolarized resonances of tyrosine (Tyr), tryptophan (Trp), 4-hydroxyphenylacetic acid (4-OH-PAA), 4-hydroxybenzoic acid (4-OH-BA), urocanic acid (UCA), homocysteine (Hcy), guanosine (Guo), and 6-hydroxynicotinic acid (6-OH-NA) are indicated. (c) Molecular structures of the metabolites together with the respective numbering scheme used for all ^{1}H nuclei. All experiments were conducted with 16 scans and a recycle delay (d1) of 3 s.

1D ¹H selective TOCSY experiments of the "urine 2" sample

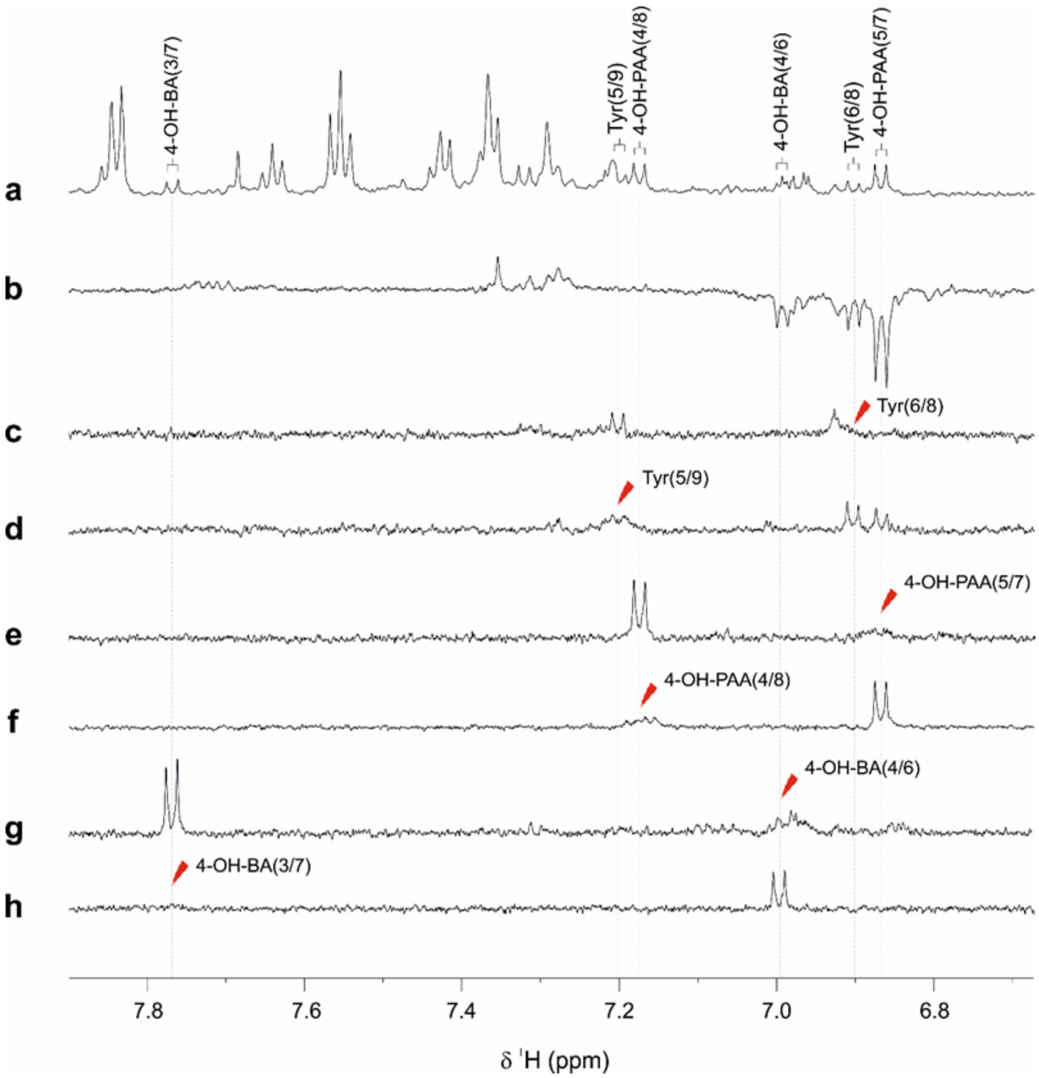

Figure S-2. Comparison of the aromatic region of several 1D ^{1}H spectra of the sample "**urine 2**". (a) Standard 1D ^{1}H, (b) 1D ^{1}H photo-CIDNP and selective 1D TOCSY spectra at irradiated frequencies of (c) 6.902, (d) 7.202, (e) 6.867, (f) 7.174, (g) 6.995, and (h) 7.769 ppm, respectively, are shown.

Thermal and photo-CIDNP spectra of the “spiked serum” sample

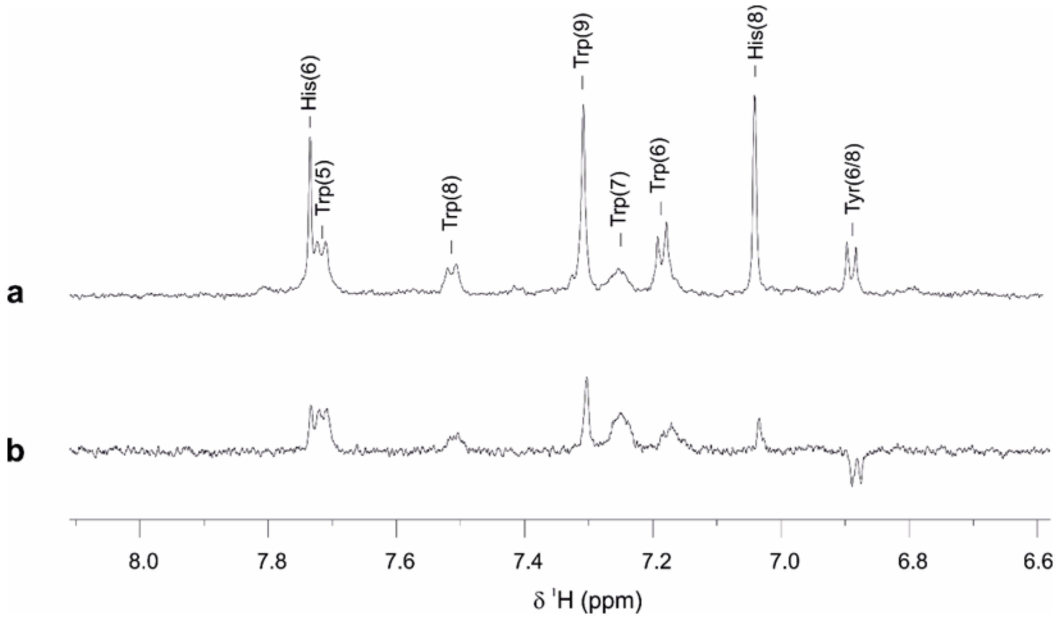

Figure S-3. Comparison of the aromatic region of a) 1D ^{1}H CPMG and b) 1D 1H photo-CIDNP NMR spectra of the “**spiked serum**” sample (Trp, 0.520 mM; His, 0.341 mM; Tyr, 0.022 mM; Met, 0.350 mM). Identification of Trp and other amino acid signals are indicated.

Evaluation of the reproducibility of the method

A preliminary evaluation of the reproducibility of the method was performed using an unaltered human urine sample ("urine 3"). The assessment was based on the results of three replicates obtained from three independent sample preparations starting from the same urine stock solution (see Materials and Methods). Previously, a standard ^{1}H spectrum of the sample showed that this specific sample of urine was significantly less concentrated. However, the 1D ^{1}H photo-CIDNP NMR spectrum showed two NMR signals, HA at 7.97 ppm and HB at 2.50ppm – corresponding to urocanic acid and homocysteine, respectively, see Figure 3 – which yielded a sufficiently high SNR to assess the reproducibility of the method (SNR > 6). Following the described workflow (Scheme S1), three replicates of 1D ^{1}H photo-CIDNP NMR spectra were obtained. Figure S4 highlights the 1D ^{1}H photo-CIDNP NMR spectrum of the "urine 3" sample as well as expanded areas showing the HA and HB signals of the three replicates (R1, R2 and R3), respectively. The absolute values of the HA and HB signal integrals were measured in each spectrum and the results were statistically analyzed (Figure S5). The coefficients of variation (CV) associated with the absolute integral values of the NMR signals range from 2 to 4%, which means that variations in metabolite concentrations of more than 4% can be detected with the described method. This value can be even lower when working with normalized peak integrals. These results demonstrate the high reproducibility of the method, which compares very favorably with other hyperpolarization methods used for the analysis of biofluids (A. Bornet *et al*., *Anal. Chem*. **2016**, *88*, 6179-6183). This high reproducibility of the photo-CIDNP NMR method presented here is not surprising given the minimal sources of variability in the experimental setup (Scheme S1).

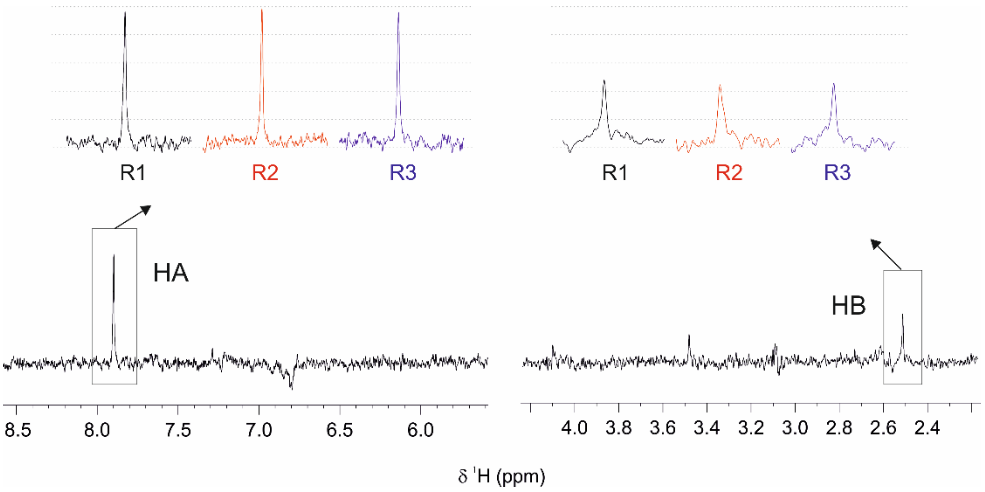

Figure S-4. Reproducibility of the method. 1D ^{1}H photo-CIDNP NMR spectrum of the "**urine 3**" sample. Proton signals at 7.97 ppm (HA) and at 2.50 ppm (HB) are highlighted, which correspond to urocanic acid and homocysteine, respectively. Expanded areas of the three replicates (R1, R2 and R3) showing HA and HB are represented.

(A)

Peak	Peak Integral (a.u.)	
	**HA**	**HB**
[^{1}H δ (ppm)]	7,97	2,50
R1	1,17E+05	4,60E+04
R2	1,14E+05	4,27E+04
R3	1,19E+05	4,34E+04
**MEAN**	1,17E+05	4,40E+04
**SD**	2,43E+03	1,74E+03
**SE**	1,40E+03	1,00E+03
**% CV (individual peaks)**	2,1	3,9

(B)

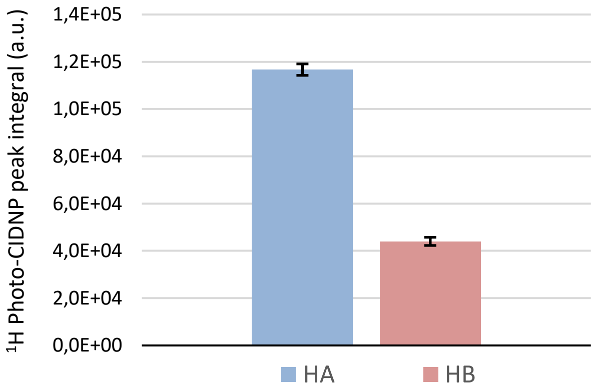

Figure S-5. Reproducibility of the method. Numerical data of the three replicates (R1, R2 and R3) of 1D ^{1}H photo-CIDNP NMR spectra. (A) Table containing the absolute values of HA and HB signal integrals for each replicate. The mean integral value and the associated standard deviation (SD), standard error (SE) and percent coefficient of variation (CV) are indicated for each NMR signal. (B) Graphical representation of mean values with error bars representing the coefficient of variation for each analyzed NMR signal.

Signal enhancement factors (ε) of hyperpolarized resonances in photo-CIDNP spectra

Table S-2. Photo-CIDNP enhancement factors (ε) of polarized NMR signals recorded in samples: “mixture 1”, “spiked urine 1”, “urine 2”, “spiked serum” and “serum”.

	M1			spiked urine 1			urine 2			spiked serum			serum			sign
H_{id}	δ (ppm)	(a)	ε	δ (ppm)		ε	δ (ppm)		ε	δ (ppm)		ε	δ (ppm)		ε	A/E
**Guo(8)**				-		(d)	8.04	(s)	(e)	-		(d)	-		(f)	A
**His(6)**	7,71	(s)	1,31 (b)	8,11	(s)	1,46	-		(d)	7,73	(s)	(c)	7,73	(s)	(e)	A
**UCA(6)**				7,98	(s,br)	(c)	7,98	(s,br)	1,16	-		(d)	-		(f)	A
**Trp(5)**	7,71	(d)	2,65 (b)	7,73	(d)	6,69	7,73	(d)	(c)	7,71	(d)	(c)	-		(f)	A
**UCA(8)**				7,36	(s)	(c)	7,36	(s)	1,28	-		(d)	-		(f)	A
**Trp(9)**	7,29	(s)	2,05	7,32	(s)	4,31	7,32	(s)	1,78	7,30	(s)	1,12	-		(f)	A
**Trp(7)**	7,26	(t)	2,59	7,28	(t)	6,73	7,28	(t)	(c)	7,25	(t)	1,39	-		(f)	A
**4-OH-PAA(4/8)**				-		(d)	7.17	(d)	(e)	-		(d)	-		(f)	A
**His(8)**	7,02	(s)	1,20	7,19	(s)	1,25	-		(d)	7,03	(s)	1,06	7,03	(s)	(e)	A
**4-OH-BA(4/6)**				-		(d)	6,99	(d)	2,16	-		(d)	-		(f)	E
**Tyr(6/8)**	6,87	(d)	1,43	6,90	(d)	5,22	6,90	(d)	2,92	6,88	(d)	1,23	6,88	(d)	(e)	E
**4-OH-PAA(5/7)**				6,87	(d)	4,73	6,87	(d)	3,45	-		(d)	-		(f)	E
**6-OH-NA(6)**				-		(d)	6,59	(d)	(e)	-		(d)	-		(f)	E
**Trp(2)**	4,03	(q)	1,16	-		(d)	-		(d)	-		(d)	-		(f)	A
**Trp(3)**	3,46	(dd)	2,06	3,49	(dd)	(c)	-		(d)	3,47	(dd)	(c)	-		(f)	E
**4-OH-PAA(2)**				3,46	(s)	(c)	3,46	(s)	1,25	-		(d)	-		(f)	A
**Trp(3')**	3,28	(dd)	(c)	3,31	(dd)	(c)	-		(d)	3,28	(dd)	(c)	-		(f)	E
**His(3)**	3,19	(dd)	1,01	-		(d)	-		(d)	-		(d)	-		(f)	E
**His(3')**	3,08	(dd)	1,02	-		(d)	-		(d)	-		(d)	-		(f)	E
**Met(4)**	2,62	(t)	1,02	-		(d)	-		(d)	-		(d)	-		(f)	A
**Hcy(4)**				2,50	(br)	(c)	2,50	(br)	1,07	-		(d)	-		(f)	A
**Met(5)**	2,12	(s)	1,02	2,14	(s)	1,03	-		(d)	2,12	(s)	(c)	-		(f)	A

(a) signal multiplicity (s, singlet; d, doublet; t, triplet; dd, doublet of doublets; br, broad signal); (b) deconvolution applied to allow measurement of the signal enhancement factor; (c) not measured due to severe signal overlap in the thermal spectrum; (d) not detected in the photo-CIDNP spectrum; (e) not detected in the thermal spectrum but detected in the photo-CIDNP spectrum; (f) not detected in neither the thermal nor the photo-CIDNP spectrum.
